# Regulating the discriminatory response to antigen by T-cell receptor

**DOI:** 10.1042/BSR20212012

**Published:** 2022-03-23

**Authors:** Kaustav Gangopadhyay, Swarnendu Roy, Soumee Sen Gupta, Athira C. Chandradasan, Subhankar Chowdhury, Rahul Das

**Affiliations:** 1 Department of Biological Sciences, Indian Institute of Science Education and Research Kolkata, Mohanpur campus, Mohanpur 741246, India; 2 Centre for Advanced Functional Materials, Indian Institute of Science Education and Research Kolkata, Mohanpur campus, Mohanpur 741246, India

**Keywords:** carbohydrate metabolism, Kinetic Proofreading, protein-tyrosine kinases, signalling, T cell receptor, zap-70

## Abstract

The cell-mediated immune response constitutes a robust host defense mechanism to eliminate pathogens and oncogenic cells. T cells play a central role in such a defense mechanism and creating memories to prevent any potential infection. T cell recognizes foreign antigen by its surface receptors when presented through antigen-presenting cells (APCs) and calibrates its cellular response by a network of intracellular signaling events. Activation of T-cell receptor (TCR) leads to changes in gene expression and metabolic networks regulating cell development, proliferation, and migration. TCR does not possess any catalytic activity, and the signaling initiates with the colocalization of several enzymes and scaffold proteins. Deregulation of T cell signaling is often linked to autoimmune disorders like severe combined immunodeficiency (SCID), rheumatoid arthritis, and multiple sclerosis. The TCR remarkably distinguishes the minor difference between self and non-self antigen through a kinetic proofreading mechanism. The output of TCR signaling is determined by the half-life of the receptor antigen complex and the time taken to recruit and activate the downstream enzymes. A longer half-life of a non-self antigen receptor complex could initiate downstream signaling by activating associated enzymes. Whereas, the short-lived, self-peptide receptor complex disassembles before the downstream enzymes are activated. Activation of TCR rewires the cellular metabolic response to aerobic glycolysis from oxidative phosphorylation. How does the early event in the TCR signaling cross-talk with the cellular metabolism is an open question. In this review, we have discussed the recent developments in understanding the regulation of TCR signaling, and then we reviewed the emerging role of metabolism in regulating T cell function.

## Introduction

**Figure 1:**
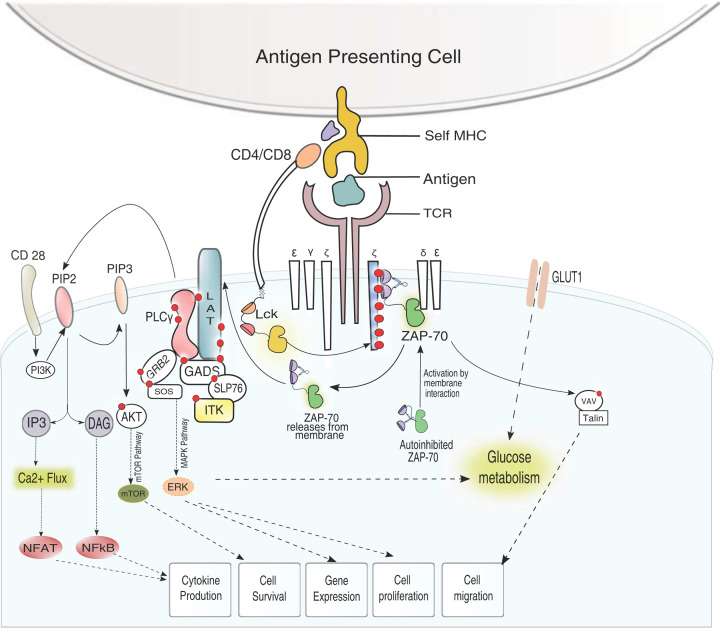
Overview of TCR signaling The key proteins known to regulate kinetic proofreading in the early TCR signaling are highlighted. Following the binding of pMHC to the TCR complex, Lck is activated and brought into proximity of the CD3 complex. Lck then phosphorylates the ITAMs in the CD3 chains (phosphorylation depicted as red dots). ZAP-70 is recruited to the TCR complex through the tSH2 domain and doubly phosphorylated ITAM interaction. Activated ZAP-70 phosphorylate scaffold protein LAT connects the TCR to indicated downstream cellular response through multiple signaling pathways. Abbreviations: ITAM, immunoreceptor tyrosine-based activation motif; LAT, linker for activation of T cells; Lck, lymphocyte-specific protein tyrosine kinase; tSH2, two SH2 domains; ZAP-70, zeta chain-associated protein tyrosine kinase.

The cell-mediated immunity is carried out by a repertoire of clonally diverse T lymphocytes that display unique antigen-binding receptors on the cell surface called T-cell receptors (TCRs). The TCR binds to the foreign antigen, presented as peptide-major histocompatibility complex (pMHC) through antigen-presenting cells (APCs) with remarkable selectivity and sensitivity (Figure 1) [1–4]. The formation of the TCR–pMHC complex initiates a cascade of downstream signaling that remodels cell metabolism and gene expression causing T cells to exit quiescence [5–8]. TCR signaling is also essential for T-cell development and maturation [9–11]. T lymphocytes migrate to the thymus gland from bone marrow to develop into mature T cells [11–13]. T cells undergo a series of selection processes in the thymus that train them to distinguish between self and foreign antigen. In the first selection step, T cells undergo positive selection to create a repertoire of self-MHC-restricted T lymphocytes [14–16]. In the subsequent steps, T cells that react too strongly with self-MHC or self-peptides are eliminated by the process of negative selection producing self-tolerant T cells [17–23].

Central to the TCR response lies a delicate balance that helps discriminate between ‘self’ versus ‘non-self’ peptides while maintaining high sensitivity against small amounts of non-self antigen (agonist). TCR does not possess intrinsic catalytic activity; it responds to antigen binding by recruiting several enzymes and adaptor proteins following a mechanism that may have evolved 500 million years ago in jawed fish [24–30]. Early TCR signaling begins with assembling coreceptors at the TCR [31–34], phosphorylation of key non-receptor tyrosine kinases [35,36], and adaptor proteins [37–39] that helps propagate the signaling downstream (Figure 1). A kinetic proofreading mechanism was proposed to explain how the lifetime of TCR–pMHC complex and subsequent delayed recruitment and activation of enzymes fine-tune the TCR downstream signaling [40–42]. In this review, we focus on the regulation of early TCR signaling. We have discussed recently published experimental evidence that explained how such a proofreading mechanism works in T cells. In recent years, it has become evident that remodeling of glucose metabolism is critical in determining the output of TCR signaling. In the final section, we discuss the emerging role of metabolic cues in regulating T-cell signaling.

## Overview of TCR signaling

TCR is a complex of integral membrane proteins comprising α and β chains, and the CD3 chains (comprising γ, δ, ε, and ζ) that together provide an extracellular ligand-binding domain and intracellular segment for recruiting enzymes and adaptor proteins (Figure 1) [7,8,43–47]. The extracellular domain of TCR interacts with the pMHC, initiating the signal transduction circuitry by recruiting coreceptors like CD4/CD8. The early downstream signaling begins with the recruitment and activation of two non-receptor tyrosine kinases to the TCR. First, a lymphocyte-specific protein tyrosine kinase (Lck) associated with the CD4/CD8 is recruited to the TCR complex [36,48,49], which in turn phosphorylates the cytosolic segment of the CD3 (γ, ε, δ, and ζ) chain. [50–55]. The phosphorylated CD3 chains serve as a docking site for the second non-receptor tyrosine kinase named zeta chain-associated protein tyrosine kinase (ZAP-70) [56–59]. The discriminative ability of TCR relies on two different events, the affinity of the antigenic peptide for the extracellular receptor [60] and the intracellular balance between downstream kinases and phosphatases, creating a feedback regulation [61–65]. Activation of Lck serves a dual role; it creates the docking site for the ZAP-70 recruitment to the membrane and fully activates ZAP-70 by phosphorylating critical tyrosine residues on the kinase domain [66–70]. Specific phosphatases, CD45, and SHP1 [36,71–74], control activation of Lck [36,75,76], which in turn regulate the recruitment of ZAP-70 to TCR. The ZAP-70 activation is central for the propagation of TCR signaling downstream (Figure 1). Together, the intricate circuitry between downstream signaling modules in the early stage of TCR signaling constitutes a proofreading mechanism making TCR sensitive to minor perturbation of antigen peptide sequence [41,77]. Next the activated ZAP-70 propagates the signal downstream by phosphorylating a scaffold protein linker for activation of T cells (LAT) [37–39]. LAT is then recruited to the signalosome where several tyrosine residues are phosphorylated by ZAP-70. An electrostatic selection mechanism filters LAT from being non-specifically phosphorylated by Lck [78,79]. Phosphorylated LAT connects the TCR signaling to cellular processes regulating cell migration, differentiation, and proliferation by recruiting enzymes and adaptor proteins to the signalosome (Figure 1) [80–83]. LAT function as a scaffold to bind adaptor proteins and enzymes like SH2 domain containing leukocyte protein of 76 kDa (SLP-76), growth factor receptor-bound protein 2 (Grb2), and phospholipase C-γ1 (PLC-γ1) connecting TCR response to the MAP kinase pathway and Ca^2+^ signaling (Figure 1). [84,85] Activated ZAP-70 also regulates T-cell migration through SLP-76 phosphorylation that connects TCR signaling to Vav1 (Figure 1) [86–88].

## Early TCR signaling is regulated by kinetic proofreading mechanism

T cells are sensitive to a very low abundance of agonist present in a large amount of self-peptide mixture. Experimentally, 60–200 molecules of the specific pMHC are sufficient for generating T-cell response [89,90]. T cells do not rely on TCR-dependent basal signaling for survival in a ligand-free state [91–93]. Deletion of TCR does not affect the T-cell survival suggesting the TCR signaling is not indispensable for cell survival in resting state. However, TCR deletion or loss of TCR affects T-cell development and maintenance [94,95]. These observations suggest that the general state of TCR is an OFF state. TCR could distinguish between an agonist and a partial agonist despite the marginal difference in the binding affinity [96–100]. The inability of the partial agonist to activate TCR downstream signaling suggests a prevalence of proofreading mechanisms that help calibrate TCR response to an agonist [101,102].

Two theories explaining how a proofreading mechanism may regulate T-cell response to antigen binding were proposed in the mid-nineties [40,103,104]. Lanzavecchia and colleagues proposed a serial triggering model suggesting that the amplification of TCR activation depends on multiple binding of the same ligand to different TCRs [103]. According to this model, a sustained TCR signaling may be generated by serially triggering large number of receptors with a limited set of pMHC molecules. For such a model to work, the ligand needs fast off rates to serially bind large number of receptors. Several computational models and experimental studies have supported the serial triggering model [105–111]. However, a direct evidence supporting serial triggering model is lacking and none of the data explained the optimal half-life of pMHC–TCR complex required to initiate an effective downstream response. Recent studies with traction force microscopy instead suggest that TCRs mechanically sense the strength of pMHC binding, and digitally activate the downstream signaling [112–116].

**Figure 2:**
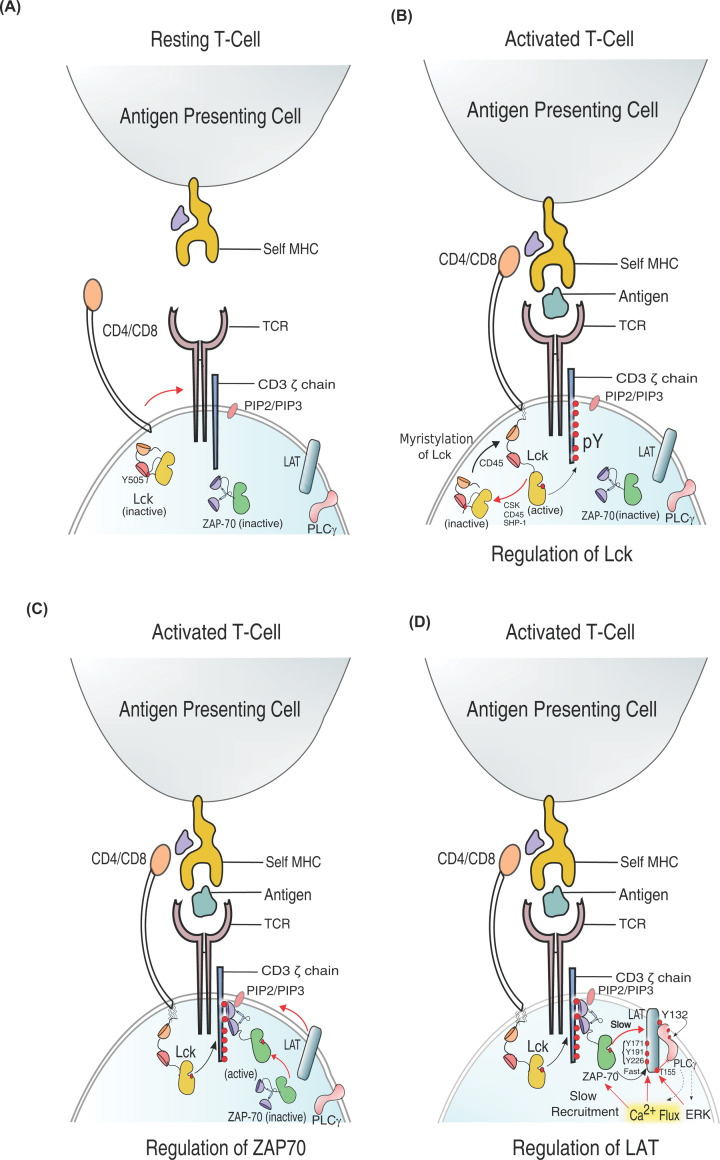
Schematic depiction of initiation of TCR signaling cascade (**A**) In the resting T cell, Lck, ZAP-70, and LAT remain in autoinhibited states. (**B**) pMHC–TCR complex formation leads to colocalization of CD4/CD8 coreceptors associated with Lck to the signalosome. (**C**) Autoinhibited ZAP-70 is recruited to the membrane and activated Lck or by autophosphorylation. (**D**) Activated ZAP-70 phosphorylates LAT and leads to the formation of LAT/SLP-76 signalosome and recruitment of PLC-γ. Red arrows indicate the rate-limiting steps.

McKeithan, in the same year as Lanzavecchia and colleagues [103], suggested an alternate kinetic proofreading model explaining TCR response on binding agonist or a partial agonist [40]. According to the kinetic proofreading model, time delays between pMHC binding to TCR and subsequent recruitment and activation of each downstream enzyme were considered. A balance between pMHC–TCR complex formation and the delayed response of downstream signaling modules determines the TCR selectivity and sensitivity. The non-specific interaction between self-antigen (or a partial agonist) and TCR are short-lived. Hence, they do not signal because the pMHC–TCR complex disintegrates before the downstream enzymes are recruited and activated [41,77,117]. Recent optogenetic approaches using chimeric ligand suggested that the half-life of optoligand and TCR complex is the rate-limiting step during the formation of the initial signalosome [41,77,117]. The ligand:receptor half-life determines the ZAP-70-dependent diacylglycerol (DAG) accumulation, an important signaling event that connects TCR to intracellular Ca^2+^ release (Figure 1) [77,117]. The interaction between foreign antigen and TCR is long-lived and finds enough time to signal by activating the kinases. Several research groups have independently studied the kinetic proofreading by TCR signaling in recent years [41,84,117–120]. The binding of TCR and pMHC acts as a driving force for the colocalization of coreceptor, bringing Lck to the close vicinity of the cytosolic domain of TCR, facilitating phosphorylation of the CD3 chains, and subsequent recruitment of other enzymes and scaffolds (Figures 1 and 2). In the following section, we discussed how such an intricate network of kinase activation is regulated during T-cell response.

## Regulation of Lck activation

Lck is an Src family kinase essential for the phosphorylation of the cytosolic domain of the CD3 chain in the TCR [121,122]. The localization of coreceptors CD4/CD8 along with Lck at the signalosome marks the activation of the first tyrosine kinase. The activated Lck phosphorylates the tyrosine residues on the immunoreceptor tyrosine-based activation motifs (ITAMs) on the CD3 chains [121,122]. The recruitment of Lck and subsequent phosphorylation of the ITAMs contribute to the temporal lag observed in the initiation of signaling cascade [123–125].

The secondary structure of Lck comprises an N-terminal regulatory module made up of SH4 domain, a unique domain (UD), SH3 and SH2 domain, connected to a kinase domain followed by a short C-terminal tail [126–130]. The autoinhibitory state of the kinase domain is stabilized by the close conformation of SH3, SH2, and kinase domain [36,131]. The autoinhibited structure is locked in the closed conformation by docking the phosphotyrosine residue (Y505) in the C-terminal tail on the SH2 domain [130]. The Y505 is phosphorylated by C-terminal Src kinase (Csk) [132–134]. Myristylation of Ser^6^ or palmitoylation of Gly^2^ in the SH4 domain is essential for membrane recruitment of Lck and subsequently releasing the autoinhibition of the kinase domain. The kinase domain inhibition is released by dephosphorylating Y505 followed by autophosphorylation of Y394 in the activation loop (Figure 2) [135–140]. Membrane-associated phosphatase CD45 helps stabilize the active conformation by dephosphorylating Y505, breaking the lock between Y505 and SH2 domain [71,141]. A delicate balance of phosphorylation and dephosphorylation regulates Lck activity. Studies using phosphomimetic mutant of Lck suggest that residue Y192 in the SH2 domain is essential in determining the equilibrium between an active and inactive conformation [131,142]. Phosphorylation of Y192 prevents Lck interaction with the CD45, thus altering the enzymatic activity of Lck and shifting the equilibrium toward the closed-autoinhibited state [143].

Apart from phosphorylation of Y192, Lck is also down-regulated by the phosphorylation of S59 in the UD domain [141,144]. S59 is phosphorylated by extracellular signal-regulated kinases (Erk1/2) [145] and dephosphorylated by calcineurin [146], creating a feedback mechanism [141]. A mutational study suggested that S59 plays a pivotal role in recruiting phosphatase SHP1, which inactivates Lck by dephosphorylating Y394 in the activation loop [147]. Conversely, studies using knock-in mutant (S59A) mice suggest a limiting role of SHP-1 interaction in favoring autoinhibited conformation of Lck during thymocyte maturation (i.e. DN to DP stage). Rather a trimeric complex between THEMIS:GRB2:SHP-1 negatively regulates Lck activation [148].

A large intracellular concentration of Lck could phosphorylate the CD3 chain in the basal state, which may initiate TCR signaling even without binding to pMHC. The basal activity of Lck is regulated by a coordinated function of non-receptor tyrosine kinase Csk and the phosphatases (CD45 and SHP-1) [147,149]. With such a large number of inhibitory steps, how does Lck initiate the signaling? The N-terminal region of Lck plays a major role in regulating Lck activation at the membrane. The N-terminal SH4 domain of Lck interacts with the Zinc-clasp motif located in the cytosolic tail of the coreceptor CD4/CD8, clustering Lck at along with CD45, an essential step for Lck-dependent ITAM phosphorylation [131,150–154]. Kinetic segregation of Lck:CD4/CD8 complex from the CD45 and Csk may result in a high concentration of active Lck accumulation at the TCR complex [131,155–158]. Recent studies suggest that antigen-bound TCR scans for several CD4/CD8 coreceptors and finally binds with CD4/CD8 coreceptors in complex with Lck [159]. The basic residues in the CD3ε chains serve as a docking site for Lck, leading to the phosphorylation of the ITAMs [160]. The clustering of Lck to the TCR may represent an additional proofreading step, most likely following a spatial proofreading mechanism [161]. Under the basal state, the Lck is spatially arranged far from the TCR in a low substrate-concentrated region. Upon TCR activation, Lck diffuse to the high substrate concentration region contributing to additional delay in activating TCR response [161].

Under the basal condition, the residual activity of Lck could partially phosphorylate ITAMs, but is not enough for activation of ZAP-70. Complete phosphorylation of the ITAMs are required for TCR engagement, which depends on the half-life of the TCR–pMHC complex [110,162]. The phosphorylation step acts as a threshold for the activation of T-cell signaling. In the basal state, the tyrosine residues in the cytosolic domain of the CD3 chain are embedded in the lipid bilayer, making them inaccessible for phosphorylation by Lck [163–165]. The binding of pMHC to the TCR reorients the cytosolic domain of the CD3 chain dislodging the tyrosine residues from the membrane allowing the UD of Lck to interact.

## Regulation of ZAP-70 activation

ZAP-70 is indispensable for propagating downstream TCR signaling. ZAP-70 is a Syk family kinase translated as a single polypeptide chain containing an N-terminal regulatory module connected through a flexible linker, interdomain-B, to the C-terminal catalytic module. The regulatory module comprises tandem repeats of two SH2 (tSH2) domains, N-SH2 and C-SH2, interconnected by a helical linker called interdomain-A [69,70,166–169]. The kinase domain adopts Cdk/Src-like inactive conformation in the autoinhibited state, stabilized by a closed conformation of kinase domain, interdomain-B, and tSH2 domain sandwich [166,170]. In the autoinhibited state, the SH2 domains are separated in an L-shaped conformation, making them incompatible with the binding doubly phosphorylated ITAM. The ZAP-70 is activated in two steps. In the first step, the autoinhibition of the kinase is partially released when the tSH2 domains bind allosterically to the phosphorylated tyrosines in the ITAM (Figure 2) [171]. Structural analysis suggests that the *holo*-tSH2 domain rearranges to a Y-shaped closed conformation exposing the Y315 and Y319 in the interdomain B to be phosphorylated [166,169,172–174]. The activated structure of ZAP-70 is stabilized by the phosphorylation of Y315 and Y319 in interdomain B, and tyrosine residue in the activation loop by Lck [169,172,173,175–178].

Steady-state ligand-binding experiments suggest that the tSH2 domain binds to the doubly phosphorylated ITAM peptides in a biphasic manner [179]. In the first step, the C-SH2 domain binds uncooperatively to the N-terminal tyrosine phosphate with low nanomolar affinity, subsequently facilitating the formation of the N-SH2 phosphate-binding pocket enabling second phosphotyrosine to bind with micromolar affinity leading to remodeling of C-SH2-binding site. Fluorescence recovery after photobleaching (FRAP) study suggests that the recruitment of ZAP-70 follows a biphasic pattern in cells [180,181]. The functional significance of such two-step ligand binding is still not completely understood. Comparison of the *apo-* and *holo-*structure of the tSH2 domain revealed that the N-SH2-binding pocket formed at the interface of the tSH2 domains [169,174,179,182]. A non-covalent network of amino acids residues allosterically couples the tSH2 domains during ligand binding. Mutation in the allosteric network residues, including W165C that cause rheumatoid arthritis-like symptoms in mice [183], uncouples the ligand-binding from ZAP-70 activation [179]. Thus, it is speculated that the ITAM binding by the ZAP-70 tSH2 domain may be an important rate-limiting step in regulating ZAP-70 activation [117,184].

Although partial phosphorylation of ITAMs allows ZAP-70 to localize on the membrane, it cannot initiate the T-cell signaling, indicating an added layer of proofreading preventing ZAP-70 activation. Mass spectrometry-based phosphoproteomics studies suggest that ZAP-70 does not activate or initiate T-cell signaling without binding to its ligand at TCR [173,175,185]. The C-terminal SH2 domain of ZAP-70 interacts with phosphatidylinositol 4,5-bisphosphate (PIP2) and PIP3 lipid in the membrane in a spatiotemporal manner priming the tSH2 domain to bind doubly phosphorylated ITAMs [186]. Subsequent phosphorylation of the tyrosine residues in interdomain B increases the retention time of ZAP-70 at the membrane [119]. The strength of TCR signaling is determined by the ZAP-70 dwell time. Following ZAP-70 recruitment to the TCR by Single-particle tracking, Lillemeier and colleagues showed that minutes (<10 min) after ITAM binding, during early T-cell activation, ZAP-70 is released from the TCR complex and translocated to the plasma membrane [118]. Mass spectroscopy analysis and immunoblot assay suggest that Y126 residue in tSH2 domain of ZAP-70 determines the half-life at the TCR. Autophosphorylation of Y126 decreases the affinity of the tSH2 domain for phosphorylated ITAM, thus releasing the ZAP-70 from the TCR complex [118,187,188]. The signaling is again initiated by recruiting new ZAP-70 molecules to the TCR complex following a ‘catch-and-prey’ model [118]. At the membrane, ZAP-70 phosphorylates the substrate peptide in the LAT with high specificity. The kinase domain of ZAP-70 carries a high net positive charge at the substrate binding site that allows specific binding of the negatively charged substrates like LAT and SLP-76 [78]. It has also been established that Lck-dependent ZAP-70 phosphorylation mediates the adaptor function of Lck, essential for bringing LAT to the TCR for activation [84,173,175,189]. The net negative charge in the substrate-binding site of Lck prevents transactivation of LAT [78].

## Propagating signal through LAT and Plc-γ

The LAT is an essential scaffold that coordinates early TCR signaling to downstream cellular responses in a phosphorylation-dependent manner. At the plasma membrane LAT and its binding partners colocalize into micrometer or submicrometer clusters [190]. Elimination of these microclusters by deleting key components (for example, LAT or Grb2) impairs downstream signaling and transcriptional responses [191].

Several phosphorylation sites on LAT are vital for kinetic proofreading TCR response to antigen binding (Figure 2). Among them, relatively slower phosphorylation of Y132 in LAT creates a critical kinetic bottleneck in transducing signaling downstream [84]. ZAP-70 phosphorylates Y132, which serves as a docking site for a PLC-γ1. The slower phosphorylation of Y132 is due to residue G131 located at the −1 position from Y132. Glycine at this position reduces the net negative charge of the peptide, making it a poor substrate for ZAP-70. Replacing G131 with an acidic amino acid accelerates the phosphorylation rate of Y132 and increases the PLC-γ1 activation, causing TCR to activate even with weak agonists or self-peptides [78,84].

ZAP-70 also phosphorylates several distal tyrosine residues on LAT (Y171, Y191, and Y226) that facilitate the recruitment for the Grb2 family of proteins, Tec family tyrosine kinase interleukin 2 inducible T-cell kinase (Itk) and SLP-76 [192–194]. The adaptor protein SLP-76 mediates the TCR response to cell migration. At the LAT complex, the SLP-76 is phosphorylated by ZAP-70, resulting in phosphorylation of Vav-1, a critical step in regulating T-cell migration [195].

Recruitment of Itk to the signalosome connects the TCR signaling to the Ca^2+^ response. At the TCR complex, Lck activates Itk by phosphorylating Y511 residue in the activation loop leading to autophosphorylation of Y180 on the SH3 domain [196,197]. Next, the activated Itk phosphorylates two tyrosine residues, 775 and 783, respectively, turning on the catalytic domain of PLC-γ1 [198,199]. PLC-γ1 cleaves PIP2 into two secondary messengers, inositol 1,4,5-trisphosphate (IP3) and DAG (Figure 1). IP3 then interacts with its cognate IP3 receptors (IP3R) on the endoplasmic reticulum (ER), inducing Ca^2+^ influx to the cytoplasm. Increased cytoplasmic Ca^2+^ activates the calcineurin by removing the inhibitory interaction with the calmodulin (Figure 1). The free calcineurin is now available to dephosphorylate cytoplasmic localized nuclear factor of activated T cells (NFAT) (Figure 1) [121]. PLC-γ1 and LAT interaction shields the phosphotyrosine residues on LAT from dephosphorylation by CD45, providing enough time to propagate downstream signaling by activating Erk through multiple pathways [200,201]. An alternate pathway mediated by a ternary complex among PLC-γ1, Pak1, and Bam32 also activates Erk, independently of LAT [202]. Erk plays a central role in connecting TCR response to the downstream gene expression (Figure 1). Additionally, Erk also functions as a negative feedback regulator of TCR signaling. The Erk phosphorylates T155 residue on LAT, thereby preventing recruitment of PLC-γ1 [203].

Immediately after the pMHC engagement, TCRs oligomerize into microcluster to which the TCR, ZAP-70, and LAT-associated signaling modules are recruited sequentially [204,205] (Figures 1 and 2). The multiple phosphorylation sites on the LAT enable cross-linking of LAT-associated signaling modules driving the microcluster formation [192,206]. In a recent study, Yi and colleagues observed a kinetic lag (delayed recruitment) in ZAP-70 binding to TCR and subsequent LAT recruitment to the ZAP-70-bound TCR complex [181]. The observed delays in recruiting the downstream signaling module may be an essential component in regulating kinetic proofreading in T cells (Figure 2). The elevated intracellular Ca^2+^ flux negatively regulates the TCR signaling by increasing the kinetic lag of ZAP-70 and LAT recruitment to the TCR microcluster.

## Metabolic regulation of TCR signaling

**Figure 3:**
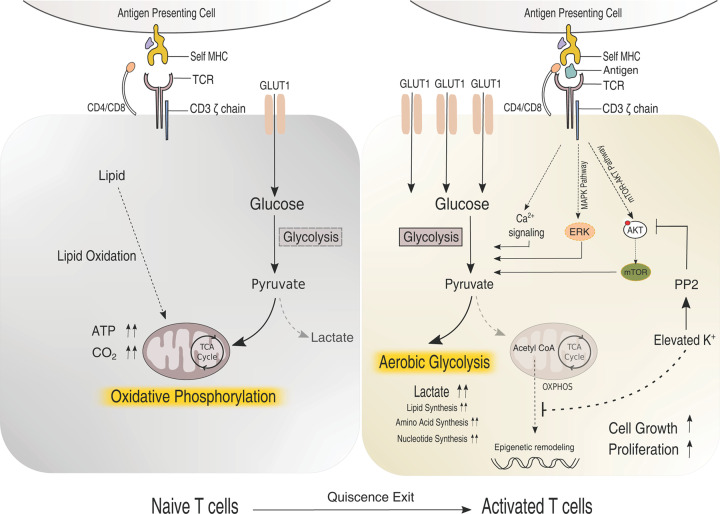
Schematic representation of T-cell metabolic network and TCR signaling The naive and activated T cells are labeled. The resting naive T cells metabolize glucose primarily via the high energy-yielding mitochondrial oxidative phosphorylation pathway. On activation, the glucose uptake is enhanced due to up-regulation of GLUT1 expression. The cells switch to aerobic glycolysis and lipid oxidation to produce biosynthetic precursors, this enhancing cell growth and proliferation. The TCR signaling modules cross-talk with the glucose metabolism are shown.

Immunometabolism has emerged as an integral regulator of immune cell responses. T lymphocytes acquire separate functional lineages on activation, and each functionally distinct states have specific metabolic requirements [207]. The naive T cells mostly remain in the quiescent stage. When activated, metabolic network is rewired to meet the demands of cytoskeletal rearrangement, clonal expansion, and epigenetic remodeling [208,209]. The naive T cell depends on lipid and pyruvate oxidation for survival [209] and converts into aerobic glycolysis and glutamine oxidation upon activation to sustain proliferation and rapid cell growth (Figure 3) [207]. The interplay between immune signaling pathways and metabolic changes is bidirectional [210]. The TCR signaling regulates metabolism, and the metabolites directly influence signaling modules or epigenetic remodeling to alter different cellular processes (Figure 3) [211]. The involvement of bidirectional metabolic signaling in regulating T-cell quiescence and activation has been extensively reviewed [208]. In T cells, the metabolite utilization is mainly regulated through the costimulatory CD28 receptor via the PI3K-Akt-mTOR pathway (Figures 1 and 3) [212–214]. TCR signaling on pMHC binding and CD28 costimulatory pathways up-regulates the expression of glucose transporter, GLUT 1, crucial for glucose uptake [215–217]. Using a genome-wide CRISPR screening and protein–protein interaction network mapping, Long and colleagues have identified key immune regulators that connect immune receptors to the nutrient-dependent downstream mammalian target of rapamycin (mTOR) signaling cascade [218]. However, the influence of early TCR signaling outputs on the metabolic network is not fully understood.

In a recent study, Jones and colleagues investigated the effect of TCR: antigen-binding affinity on the metabolic output in T cells [160,219]. Studying peptide-human leukocyte antigen (pHLA) and TCR binding, they concluded that the most robust ligand interaction might result in the highest glycolic change and hexokinase expression. Downstream TCR signaling modules play a crucial role in cross-talk with the metabolic network. Erk, which is activated downstream of the TCR signaling, regulates glucose utilization by enhancing hexokinase gene expression [220,221]. However, if hexokinase is a direct phosphorylation target of Erk is unknown. Alternatively, hexokinase II (HK-II) activation is also regulated by Akt [222,223]. Dynamic localization of HK-II between the cytosol and mitochondria is important for maintaining cellular energy balance (i.e. catabolism *vs* anabolism). Phosphorylation of HK-II by Akt promotes localization of HK-II to the mitochondria leading to increase glycolytic flux and catabolism. Together, these observations suggest that both Erk and phosphatidylinositol 3-kinase (PI3K)-Akt pathways may synergistically regulate hexokinase-dependent glucose metabolism upon TCR activation (Figure 3). Calcium ion (Ca^2+^) is another signaling modulator downstream of TCR that regulates the metabolic network of T cells. For example, a defect in the store-operated Ca^2+^ entry (SOCE) pathway, which influences Ca^2+^ flux in T cells, inhibits the phosphorylation of Akt and nutrient sensing through the mTOR pathway [224].

Elevated potassium ion concentration in the tumor microenvironment is also linked to Ca^2+^ homeostasis, which reprograms glycolysis and suppresses T-cell function. Increased potassium ion down-regulates Akt activation through serine-threonine phosphatase PP2A-dependent manner, affecting the nutrient-sensing mTOR pathway and down-regulating glucose uptake [225]. The reduction in nutrient uptake forces T cells to enter a functional caloric restriction state, thus driving mitochondrially dominant metabolism. Low nutrition leads to scarcity of cofactors like nucleocytosolic acetyl–coenzyme A limiting the acetylation of histone H3, required to promote effector functions. Depletion of methionine intermediates under starvation lowers the methylation of Histone H3 is linked to T-cell stemness [226]. Together, these observations indicate that extracellular and intracellular nutrient levels can significantly impact T-cell functioning. Since the TCR-induced immunological cues and metabolic cascades are intricately connected, reprogramming metabolic pathways is a promising tool for improving T cell-based immunotherapies [227].

## Summary

Tight regulation of signaling pathways is imperative for an accurate immune response at the right time. The adaptive immune system evolved from a lymphoid cell-based systems in jawless vertebrates to a robust BCR-TCR-MHC immune system in jawed vertebrates [25,30,228–230]. The humoral and cell-mediated parts of the adaptive immune system are governed by B and T cells, respectively [231,232]. Both the cell types share the same lineage, and the same family of proteins mediate the receptor-dependent downstream signaling following a conceptually similar mechanism [231,233]. For example, like T cells, the early receptor signaling in B cell is initiated by Src and Syk family of non-receptor tyrosine kinases [234]. B-cell receptor signaling begins with Syk kinase recruitment, corresponding to ZAP-70 activation in TCR signaling. Despite the high sequence conservation between Syk and ZAP-70, the functional significance of the subtle difference between the two proteins in determining the respective cellular response is not clearly understood [234–236]. Chronic lymphocytic leukemia (B-CLL) aberrantly expresses ZAP-70, remodels the Syk-mediated BCR downstream signaling. ZAP-70 diverts the B cells to undergo tonic PI3K signaling and ensures cell survival, promoting malignancy [237,238].

Deregulation of crucial signaling modules in TCR signaling pathways (Figure 1) often associated with human diseases related to cellular anergy, immunodeficiency or autoimmune diseases (summarized in Table 1). Over the years, immunotherapy and immunomodulators have evolved as a potent therapeutic strategy to treat autoimmune disorders and cancer [227]. The ability of T cells to identify tumor antigens and drive antitumor activities makes adoptive cellular transfer (ACT) therapy an essential clinical approach in multiple cancer treatments [239]. Promising clinical approaches of using immunotherapies like chimeric antigen receptor T cells (CAR T) therapy, TCR engineered T-cell therapy (TCR-T), and tumor-infiltrating lymphocytes (TILs) in mitigating multiple viral, autoimmune, and malignant diseases are being developed [240–243]. Recent studies on SARS-CoV-2 patients revealed that elevated glucose levels and glycolysis facilitate increased viral replication and cause monocytes-driven cytokine storms, resulting in T-cell dysfunction [244,245]. Together these observations underline the importance of investigating intricate networks and cross-talk between metabolic pathways and immune signaling to understand the regulation of the adaptive immune system.

**Table 1:** Diseases associated with defects in early T-cell signaling candidates

Candidate protein	Regulation	Disease
Lck	Mislocalization of Lck	Thymoma [246]
	Impaired Lck inhibition	Acute coronary syndrome [247]
	Missense mutation c.1022T>C	New form of T-cell immunodeficiency [248]
CD3ζ	Reduced expression	Renal cell carcinoma [249]
	Reduced expression	Colorectal carcinoma [250]
	Reduced expression	Rheumatoid arthritis (RA) [251]
	Reduced or lack of expression	Systemic lupus erythemetosus (SLE) [252]
ZAP-70	Abnormal ZAP-70 expression in B cells	Chronic lymphocytic leukemia (CLL) [237]
	Deficiency	Severe combined immunodeficiency (SCID) [236]
	W163C mutation (mice)	Rheumatoid arthritis in SKG mice [183]
	R192W, R360P mutation	Undescribed human ZAP-70-associated autoimmune disease [253]
	P80Q/M572L	CD8+ lymphopenia [254]
	L337R	Secondary hemophagocytic syndrome [255]
	D521N	Immune thrombocytopenic purpura [256]
	c.1623 + 5G > A	Epstein–Barr virus (EBV) lymphoproliferative disease (LPD), Hemophagocytic lymphohistiocytosis (HLH) [257]
LAT	Up-regulation	Sezary syndrome [258]
	Frameshift mutation (c.44_45insT;p.Leu16AlafsX28)	T^−^B^+^NK^+^ SCID [259]
	Y136F mice	Lymphoproliferative syndrome [260]
ITK	Deficiency and R29H mutation	EBV-associated lymphoproliferative disease [261]
PLC-γ1	PLCγ1-deficient mice	Peripheral T-cell lymphopenia [262]

## Abbreviations

DAG: diacylglycerol
Erk: extracellular signal-regulated kinase
Grb2: growth factor receptor-bound protein 2
HK-II: hexokinase II
IP3: inositol 1,4,5-trisphosphate
ITAM: immunoreceptor tyrosine-based activation motif
Itk: interleukin 2 inducible T-cell kinase
LAT: linker for activation of T cells
Lck: lymphocyte-specific protein tyrosine kinase
mTOR: mammalian target of rapamycin
PI3K: phosphatidylinositol 3-kinase
PIP2: phosphatidylinositol 4,5-bisphosphate
PLC-γ1: phospholipase C-γ1
pMHC: peptide-major histocompatibility complex
SLP-76: SH2 domain containing leukocyte protein of 76 kDa
TCR: T-cell receptor
tSH2: two SH2 domains
UD: unique domain
ZAP-70: zeta chain-associated protein tyrosine kinase
